# Embodied, visible, and courteous: exploring robotic social touch with virtual idols

**DOI:** 10.3389/frobt.2024.1240408

**Published:** 2024-03-25

**Authors:** Yuya Onishi, Kosuke Ogawa, Kazuaki Tanaka, Hideyuki Nakanishi

**Affiliations:** ^1^ Interaction Science Laboratories, Advanced Telecommunication Research Institute International, Sorakugun, Japan; ^2^ Department of Mechanical Engineering, Osaka University, Suita, Japan; ^3^ Faculty of Information and Human Sciences, Kyoto Institute of Technology, Kyoto, Japan; ^4^ Faculty of Informatics, Kindai University, Higashi-osaka, Japan

**Keywords:** handshake, social touch, haptic devices, virtual interaction, human-likeness, virtual idol

## Abstract

In recent years, virtual idols have garnered considerable attention because they can perform activities similar to real idols. However, as they are fictitious idols with nonphysical presence, they cannot perform physical interactions such as handshake. Combining a robotic hand with a display showing virtual idols is the one of the methods to solve this problem. Nonetheless a physical handshake is possible, the form of handshake that can effectively induce the desirable behavior is unclear. In this study, we adopted a robotic hand as an interface and aimed to imitate the behavior of real idols. To test the effects of this behavior, we conducted step-wise experiments. The series of experiments revealed that the handshake by the robotic hand increased the feeling of intimacy toward the virtual idol, and it became more enjoyable to respond to a request from the virtual idol. In addition, viewing the virtual idols during the handshake increased the feeling of intimacy with the virtual idol. Moreover, the method of the hand-shake peculiar to idols, which tried to keep holding the user’s hand after the conversation, increased the feeling of intimacy to the virtual idol.

## 1 Introduction

Virtual idols are nonphysical fictitious idols whose appearances are generated by computer graphics. Virtual idols can be classified into two primary types. First, the agent type in which creators write algorithms to control the movements and voices of the virtual idols, which are thereafter performed autonomously (Lam, 2016; Yin, 2018). Second, the avatar-type in which human actors perform their movements and voices (Choudhry et al., 2022). In recent years, avatar-type virtual idols have attracted increasing attention. The movement of the avatar is generated based on the actor’s facial and body movements. This type of virtual idols utilizes performances from live video streams or recorded videos. A past study reported that avatars and real individuals are streaming similar contents (Lu et al., 2021). In addition, the activities of the avatar-type virtual idols include interaction with the fans, similar to Japanese idols who perform activities such as singing, dancing, and live performances displaying their charm. For instance, in certain events, idols engage with their fans in a simple conversation (Galbraith, 2012; Yakura, 2021), which raise the feeling of intimacy between the fans and their idols.

The distinction between virtual idols and Japanese idols is the possession of a physical body. Japanese idols can conduct face-to-face communication with fans, e.g., handshake and gestures. However, virtual idols cannot easily perform these physical interactions, which limit their modes of communication. Although certain face-to-face events have been organized by virtual idols, they primarily adopted nonphysical interactions, i.e., conversations and capturing pictures. Therefore, embodying virtual idol’s body can enhance the interaction methods involving social touch between the idols and fans using hands. The purpose of this study is to raise the feeling of intimacy toward the virtual idol by replicating the social touch between Japanese idols and fans utilizing a robotic hand.

Past study reported that touch by a robotic hand influences the perception of agents regarding their cognized co-presence, and it did not influence the likability (Hoppe et al., 2020). This study applied a short tap on the participant’s shoulder as the social interaction. However, the studied scenarios did not involve the virtual idols shake hands with fans. Other study reported that an advanced controller enabled a user wearing a head-mounted display to engage in a physical handshake with a virtual agent (Wang et al., 2011). Despite this controller demonstrated physical behavior similar to a human handshake, the aspect of intimacy toward the virtual agent remained unexplored. In other handshake-related studies developed a robotic hand and attached it to a video-conference device. This system can embody a part of a remote person’s body and enhance the social telepresence (feeling as if users are meeting each other in the same room) by performing a handshake with a remotely connected individual (Nakanishi et al., 2014). Furthermore, another study reported that positional consistency between the video and the robotic hand in mobile video calls enhances social bonding and intimacy with the remote partner (Tanaka et al., 2021). Although this system was developed for remote communication, it can be applied to the embodiment of virtual idols, which is the purpose of the current study. Therefore, there is a possibility that a handshake with the virtual idol can raise the intimacy felt by a user. This study improves the robotic hand for a virtual idol and explored the feeling of intimacy towards to a virtual idol by imitating the handshake behavior of Japanese idols in a virtual idol.

Our paper is structured as follows. After introducing related work in Section 2, we describe the experiments in Section 3. Since the experiment consists of three parts, the overall goal is shown in Section 3.1. Section 4 describes the deployment, and Section 5 discusses about the results obtained from Section 3 and the feedback obtained from Section 4. And we conclude by describing our main findings in Section 6.

## 2 Related works

### 2.1 Social touch interface in human-human communication

Social touch has been extensively studied in terms of human-robot interaction, virtual environments, and remote communications. In a remote communication scenario, previous study added a haptic stimulus functionality to the existing media. For instance, a user’s mobile device vibrates when a remote partner squeezes his/her device (Chang et al., 2002). Another study adds stimuli, which can exert a poking force on cheek through a pneumatically inflated silicone balloon (Park et al., 2013). Moreover, mediate kissing device reproduced the pressure and movement of the partner’s lips using servomotors (Samani et al., 2013). This device creates a more intimate interaction than ordinary video calls. To reproduce hugs, clothing-type devices have been proposed, which simulate a conversation while viewing a mirror that displays the hugging users (Vetere et al., 2005; Morikawa et al., 2006; Teh et al., 2008; Zhang et al., 2015). Furthermore, a hug can be reproduced via a voice call while hugging a bolster with a built-in mobile phone (Sumioka et al., 2013). To reproduce a handshake a band-type device tightens the user’s hand and presents a body temperature (Yarosh et al., 2017). This device tightens each other’s hands if the users place their hands on the corresponding place. Another approach has been proposed to reproduce a handshake by attaching a human-like robotic hand with a video-conferencing terminal. These devices enhanced social telepresence (Nakanishi et al., 2014) and social bonding (Tanaka et al., 2021). In general, all these devices have been explored for human–human communication but not for agent/avatar–human communication.

### 2.2 Social touch with agents or avatars

Past studies have explored the action and effects of touch with agents or avatars. These studies can be categorized into touch in virtual environments and touch in real environments. In the case of touch in virtual environments, users exist as avatars in the virtual space and touch another avatar. Previous studies focused on the influences of the perceived impression by touch with visual (Biocca et al., 2001; Swidrak et al., 2019; Nagamachi et al., 2020; Sykownik et al., 2020; Fusaro et al., 2021; Sugimori et al., 2021) and audio stimuli (Biocca et al., 2002). For instance, a novel avatar-tracking controller enabled rapid, accurate tracking with flexible motion in response to contact (Sugimori et al., 2021). However, this study only investigated the visual and audio effects without any touch. Thus, various devices have been proposed to achieve physical touch. For instance, simple haptic gloves or arm straps have been widely used to study the touch interaction effects in virtual environments (Haans et al., 2009; Huisman et al., 2013; Huisman, 2017; Boucaud et al., 2019). Other studies employed physical robots to reproduce more realistic tactile stimuli for users in virtual environments (Kotranza et al., 2009; Wang et al., 2011). However, such physical touch aims to immerse users in virtual environments and prerequisites wearing a dedicated device.

In the case of touch in real enviroments, humanoid robots are used as agents and avatars. Previous studies reported the effectiveness of social touch with robots (Knapp et al., 2010; Bevan et al., 2015; van Erp et al., 2015; Shiomi et al., 2016; Shiomi et al., 2019; Geva et al., 2020; Shiomi et al., 2020). The robot’s touch encourages motivation (Shiomi et al., 2020), persuasion (Shiomi et al., 2016), self-disclosures (Bevan et al., 2015), and increases pain or stress-buffering effects (Shiomi et al., 2019; Geva et al., 2020). The touch interaction with robots yields positive impressions (Willemse et al., 2019) and conveys various emotions by altering the touch characteristics (Teyssier et al., 2020; Higashino et al., 2021; Zheng et al., 2020; Shiomi et al., 2020, Began et al., 2015). For instance, in a study exploring a robot’s touch on the rear side of a human hand, the length and type of touch influenced the perception of the intensity and ingenuity of the emotions expressed by the robot (Zheng et al., 2020). More importantly, a handshake with a robot used for avatar can be effective in negotiation contexts (Began et al., 2015). However, agents and avatars in these studies were not investigated for special roles (i.e., like idols). In this study we explored the effectiveness of social touch with avatars embodying a special role as the idol.

### 2.3 Touch interactions with idols

Past study has explored touch interactions with idols, epitomized by handshake events initiated by Japanese groups (Galbraith, 2017). Fans must purchase CDs that come with tickets to participate in these handshake events. And they spend a short time engaging in handshakes or high-five and conversations with specific members (Patrick and Jason, 2012; Maneechaeye, 2021). In addition, photo-session events where fans can take an instant photo with idols are also common (Lukacs, 2015). On the other hand, since virtual idols are unable to engage in physical interactions, face-to-face events have primarily utilized non-physical forms of interaction, such as conversations and photo-sessions. Another study examined the effects of combining a photo of a Japanese idol with a handshake device (Kumagai and Nonomura, 2023). It demonstrated that viewing a photo of the idol’s face placed above the handshake device while grasping it increased her perceived attractiveness. However, this study lacked interactive elements such as conversation. This study attempted to replicate the face-to-face interactions of Japanese idols, such as conversations and handshakes, in virtual idols by utilizing a robotic hand.

## 3 Experiments

### 3.1 Goal

This study aimed to raise the feeling of intimacy toward the virtual idol by replicating the social touch between Japanese idols and fans utilizing a robotic hand. To this end, we separately conducted three investigations step-by-step. First, we tested the effectiveness of a robotic handshake by comparing the proposed method that reproduces the handshake of a Japanese idol with a conventional method of face-to-face interaction with the virtual idol. Second, we considered a situation where the user continues to hold hands with the virtual idol throughout the conversation. This situation provides fans with an experience like a date. Therefore, we simulated a date continuously holding hands with a virtual idol. In our propose method, the user will see the virtual idol’s appearance including the handshake device, when the user and the virtual idol face each other while holding hands. However, a partition between the display portrayed the upper body of the virtual idol and the robotic hand, which bears a visual contradiction between the virtual space and physical space. Accordingly, we tested whether the users accept this visual contradiction. Third, we reproduced the handshake with a real idol. The handshake between a real idol and fans vary from that between general users. Real idols strive to courteously shake hands with their fans because they cherish the interaction their fans. Thus, we tested this courteous behavior in the handshake with the virtual idol.

We conducted these three investigations in laboratory experiments. The virtual idols featured in these experiments were created by a free license software. Therefore, participants met with the virtual idol for the first time. On the other hand, typical virtual idols continuously communicate with their fans, who may feel a sense of intimacy towards them. We deployed these experiments into a handshake event, and gathered feedback by observing fan behaviors.

### 3.2 Hypothesis

Japanese idol handshake events permit fans to spend a brief period with a particular member, engaging in handshakes and conversations with the idol (Maneechaeye, 2021). On the other hand, in the face-to-face event with virtual idols, the typical touch-based interaction is high-five. Fans can touch the virtual idol by touching the display. Our proposed method can reproduce the handshake of the Japanese idols. We tested the effectiveness of a robotic handshake by comparing the proposed method with a conventional method of face-to-face interaction with the virtual idol. Therefore, we formulated the following hypothesis.

Hypothesis 1-a Handshaking with an embodied hand of a virtual idol improves the feeling of intimacy towards the virtual idol than video-based interaction.

Not limited to virtual idols, real Japanese idols ask fans to purchase CDs, participate in events, and engage in other activities (Galbraith, 2012; Maneechaeye, 2021). A past study investigated the relationship between such requests and social touch (Higashino et al., 2021), reporting that social touch improved the time of task while performing them. If an idol requests fans, it may be placed during the social touch as well as after the social touch. In this experiment, we adopted requests from the virtual idol after doing a social touch. The user’s actions were performed after the request from the virtual idol. Therefore, we assessed the enjoyability of a task as a factor of motivation and formulated the following hypothesis.

Hypothesis 1-b Handshaking with an embodied hand of a virtual idol provides a sense of joy to the task after it than video-based interaction.

Conversing while holding hands with an idol provides fans with an experience like a date. Therefore, we simulated a date situation continuously holding hands with a virtual idol. If the user and the virtual idol face each other while holding hands, the user will see the virtual idol’s appearance including the handshake device. However, a partition between the display portrayed the upper body of the virtual idol and the robotic hand, which bears a visual contradiction between the virtual space and physical space. A previous study investigating video-conferencing and embodiment of the remote partner’s body clarified that this partition caused a negative impression on the user (Onishi et al., 2016). Thus, we considered that the boundary between the image of the virtual idol and robotic hand should be concealed. A previous study indicated that viewing a photo of a Japanese idol’s face placed above a handshake device, while simultaneously grasping the device, increased her perceived attractiveness (Kumagai and Nonomura, 2023). The handshake in this study employed a grip handle, concealed by fabric to render it invisible to the user, thus enabling users to engage in the handshake while imaging that they were directly holding her hand. Other studies reported that the utilization of solely audio and tactile channels can augment communication, even on devices without video channels. ComTouch augmented remote communication by converting hand pressure into vibrational intensity between users in real-time (Chang et al., 2002). POKE improved the communication using calling devices that reproduced the voice and touch channels for couples in long-distance relationships (Samani et al., 2012). According to these studies, there have a possibility that users can imagine virtual idols through voice and touch channels. Accordingly, we formulated the following hypothesis.

Hypothesis 2 Stimulating the imagination of the virtual idol’s appearance by holding hands without directly viewing them improve feelings of intimacy towards the virtual idol more than holding hands with viewing their image directly.

Handshakes between Japanese idols and fans differ from those among general users. A handshake between general users is unaffected, regardless of which user first releases the hand. However, at handshake events of Japanese idols, the idols do not release the hand first, because fans may feel rude if the idols first release their hand. Thus, idols should communicate courteously with their fans. Instead, event staff will announce the end of a handshake. Therefore, we focused on the variation in a user’s impression depending on the timing of releasing the robot hand at the end of handshake with the virtual idol. Accordingly, we formulated the following hypothesis.

Hypothesis 3 The courteous handshake from the virtual idol provides a sense of intimacy to the users.

### 3.3 Setups

#### 3.3.1 Virtual idols

We used a free license software to generate the image of virtual idols. The gender of the virtual idol was opposite to that of participants. The movement of the virtual idols was synchronized with the movement of the experimenter’s mouth and upper body, as captured through motion tracking. The voice of the virtual idols was synthesized voices, and the conversation script was prepared during the experiment. Therefore, the experimenter who was the performer of the virtual idol, lip-synced according to the conversation.

#### 3.3.2 Robotic handshake with virtual idols

Based on a previous study (Nakanishi et al., 2014), this study employed a robotic hand that has three features. First, this robotic hand reproduces substantial grip force. Specifically, wires are attached to the inside and outside of each finger. The servomotor pulls the inside wire to bend the finger and pulls the outside wire to stretch the finger. Second, the robotic hand reproduces the body temperature as a human hand. To warm the entire hand, heating cords have been placed near the fingers and palm. Third, to represent the softness of a human hand, the fingers and palm of the robot hand were coated in urethane gel, which imparts the required softness. In addition, the size of the robotic hand was evaluated based on the average size of adult hands. This robotic hand can be operated by an experimenter.

#### 3.3.3 Environments

**FIGURE 1:**
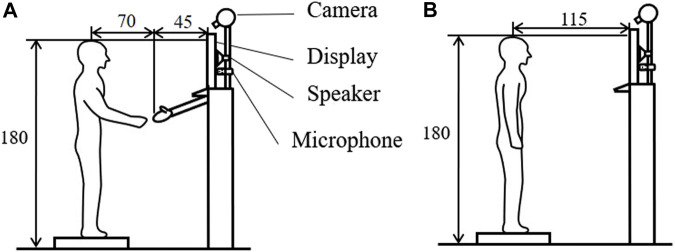
Structure of the experiment room (length unit: centimeters). **(A)** With handshake; **(B)** Without handshake.

The structural setup of the experimental room is presented in Figure 1. As depicted in Figure 1A, the robotic hand was attached to the virtual idol device under the display, acting as the virtual idol’s right arm. In Experiment 1-a and Experiment 1-b, we compare the results obtained with/without the robotic hand. As portrayed in Figures 1B if the participants and the virtual idol did not shake hands, we removed the robotic hand.

We used a 50″wide-screen display to project a nearly life-size picture of the virtual idol’s upper body, which followed the movement of the experimenter’s body. In particular, the participants stood on a platform base with adjustable height that ensured correspondence between the participant’s and virtual idol’s eye level. Additionally, the speaker installed behind the display transmitted the voice of the virtual idol. Moreover, the experimenter can view and listen to the participants via the camera and microphone. In Experiment 1-b, we placed a desk adjacent to the virtual idol device. In Experiment 2, we projected an image on the wall that was opposite to the virtual idol device.

### 3.4 Conditions

We conducted two experiments to test if the virtual idol should embody social touch. In Experiment 1-a corresponding to hypothesis 1-a, we measured the intimacy toward the virtual idol, and in Experiment 1-b corresponding to hypothesis 1-b, we assessed the joy felt in responding to a request from the virtual idol. In Experiment 1-a, we compared under the following three conditions.

• Nontouch condition: Participants engaged in a conversation without any social touch.
• Disembodied touch condition: Participants can high-five the display at the beginning and end of the conversation. The virtual idol was not embodied and the participants can touch only the display.
• Embodied touch condition: Participants shake hands with the robot hand at the beginning and end of the conversation. The virtual idol was embodied by the robotic hand and the participants can touch the virtual idol’s hand.

In Experiment 1-b, to evaluate the effectiveness of the embodied touch condition, we disregarded the Disembodied touch condition and compared the following two conditions.

• Nontouch condition: Participants engaged in a conversation without any social touch.
• Embodied touch condition: Participants shake hands with the robotic hand at the beginning and end of the conversation.

We conducted Experiment 2 to the effect of visibility with social touch corresponding to hypothesis 2. We compared under the following two conditions.

• Visible condition: Participants engaged in handshake and conversation with the virtual idol. Participants can view the boundary between the image of the virtual idol and robotic hand.
• Imaginable condition: Participants engaged in handshake and conversation with the virtual idol. Participants viewing the same direction as virtual idols but cannot observe the boundary between the image of the virtual idol and robotic hand.

We conducted Experiment 3 to the effect of courteousness with social touch corresponding to hypothesis 3. We compared under the following two conditions.

• Business-like condition: Participants and virtual idol release hands simultaneously.
• Courteous condition: The virtual idol continues to hold the participant’s hand even if the participant attempts to release their hand.

### 3.5 Procedure

In all experiments, we set a task for participants to engage in conversations with the virtual idols regarding certain locations. The virtual idol talked about various places in each condition (e.g., aquarium, zoo and museum). To conduct a disciplined experiment, the length of all conversations, number of questions, and number of responses remained constant. In particular, we reduced the number of responses during the conversation, because simple conversations with the virtual idol can produce a ceiling effect.

Participants shake hands with the virtual idol at the beginning and end of the conversation, or during the conversation. These differences depend on the procedure of the experiment. The participants were not accustomed to the action of handshake with virtual idols, and thus, they practiced the required prior to the experiment. After the task, the participants answered a questionnaire and an interview.

#### 3.5.1 Effects of embodied social touch (Experiment 1)

In Experiment 1-a, high fives and handshakes were performed at the beginning and end of the conversation. In Experiment 1-b, the virtual idol needs to make requests to participants. Therefore, we added a simple drag-and-drop task, which has earlier been used to assess the degree of effort toward a form of reward in social psychology (Hoppe et al., 2020). In this task, a red circle and a black square were projected on the display. The participants dragged the circle and dropped it into the square. After dropping the circle into the square, the circle disappears and a new circle appears at random positions on the *x*-axis. Participants repeatedly performed this operation for 3 min. First, we set a practice phase for 15 s to ensure the understanding of the task among the participants. Thereafter, the participants pressed the start button and performed the task for 3 min. After completing the task, the participants conversed with the virtual idol. Under certain conditions, the participants and virtual idol shook hands at the beginning and end of the conversation. After the conversation, the participants performed the same task for 3 min.

#### 3.5.2 Effect of visibility with social touch (Experiment 2)

In this experiment, the participants may encounter difficulty in grasping hands if they release the robot hand and attempt to grasp it again, at the imaginable condition. Therefore, we adopted the method of holding hands during the conversation instead of a handshake in this experiment. In addition, to simulate the sensation of holding hands and conversing with a virtual idol, a projector cast an image of a road scene onto the wall opposite the display.

#### 3.5.3 Effect of courteousness with social touch (Experiment 3)

In this experiment, participants were holding hands throughout the conversation with the virtual idols during the experiment. The flow of the experiment is presented in Table 1. At the end of the conversation, the virtual idol stated, “Now, we are ending the conversation,” and participants released the virtual idol’s hand. After this announcement, the virtual idol became silent. In a business-like condition, the participant and the virtual idol released hands simultaneously. In a courteous condition, the robotic hand continued to hold the participant’s hand even if the participant attempted to release the virtual idol’s hand. Consequently, in certain cases, the participants forcibly released the virtual idol’s hand. The mean of the time from the announcement of the end of the conversation to the release of the robotic hand by the participant was 2.4 s (*S.D.* = 0.2 s) in the business-like condition and 5.4 s (*S.D.* = 1.2 s) in the courteous condition. We conducted a *t*-test for these times and significant variations were observed between the conditions (*t*(20) = 2.086, *p* < .05). Therefore, the duration for which the participants held the robotic hand varied.

**TABLE 1:** Variations between time to release participants hand.

Business-like condition		Courteous condition	
Images when participant releases the virtual idol’s hand	Status of a handshake (Participant/Virtual idol)	Images when participant releases the virtual idol’s hand	Status of a handshake (Participant/Virtual idol)
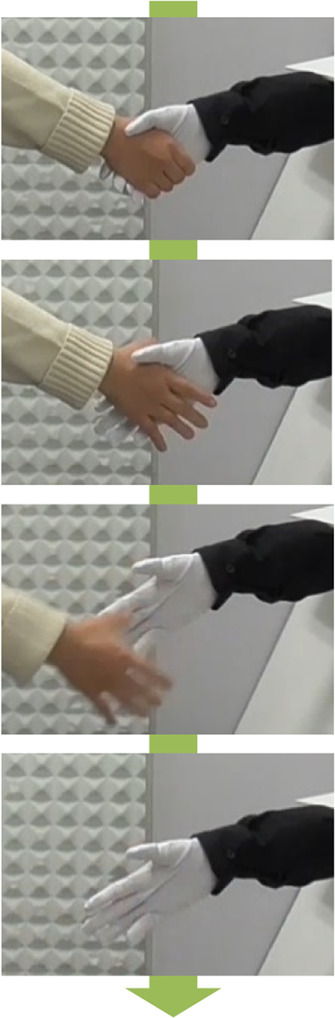	Close/CloseOpen/OpenOpen/Open-/Open	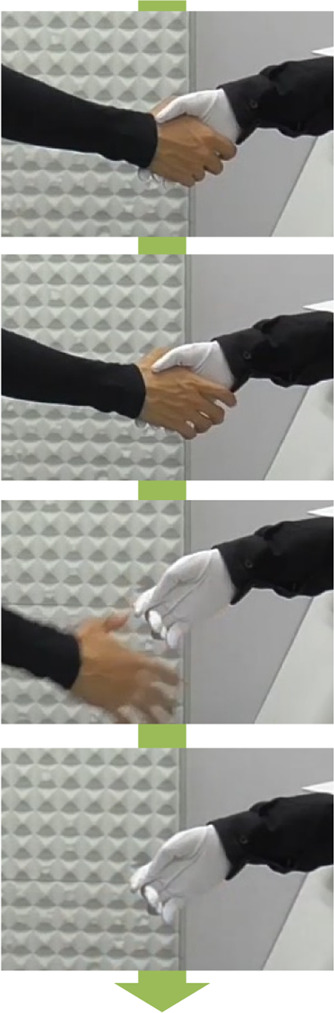	Close/CloseOpen/CloseOpen/Close-/Close
	
	
	

### 3.6 Questionnaire

After completing each task, the participants answered a questionnaire and an interview. In the Experiment 1-a, 2, 3, we asked “I felt intimacy toward the partner.” And in the Experiment 1-b, we asked “I felt that the drag-and-drop task was enjoyable”. The statements were rated on a Likert scale with lowest = strongly disagree and highest = strongly agree. In addition, the participants described the reasons for the responses in the free description.

### 3.7 Participants

Participants were undergraduate students whose ages ranged from eighteen to 24 years. Eighteen males participated in the Experiment 1-a. Recently, several situations have been reported in which female virtual idols and male fans communicate with each other. Thus, we conducted the experiment with female virtual idols and male participants, following the within-subject design. Twenty-eight individuals, consisting of fourteen females and fourteen males, participated in the Experiment 1-b, following the between-subject design. Twelve individuals, consisting of six females and six males, participated in the Experiment 2, following the within-subject design. Twenty-two individuals, consisting of eleven females and eleven males, participated in the Experiment 3. This experiment was between-subjects and had two conditions, so eleven individuals participated in each condition. In total, seventy individuals participated in our experiments.

This experiment was based on an experiment plan that was approved by Osaka University’s Research Ethics Committee (Number: 28-5-0). Participants were given a consent form before the experiment began. We conducted the experiment only if they agreed.

### 3.8 Results

**FIGURE 2:**
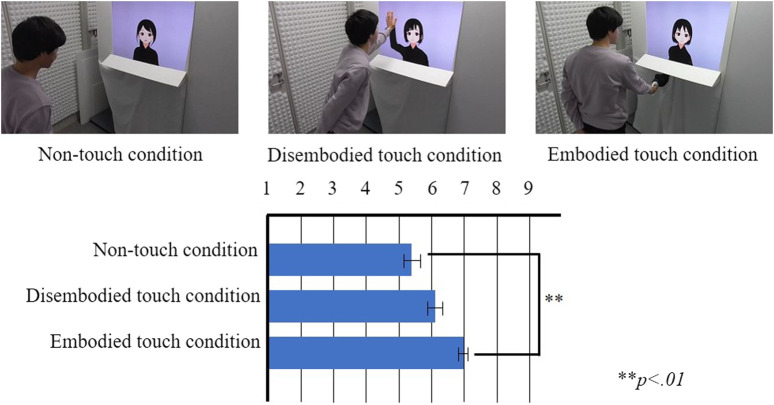
Results of the Experiment 1-a questionnaire “I felt intimacy toward the partner.”

The results of Experiment 1-a are shown in Figure 2, wherein each box represents the mean value of the responses to each statement and each bar represents the standard error of the mean value. The results indicate a comparison of the three conditions through one-way factorial ANOVA followed by Bonferroni’s test. We determined a significant variation in the feeling of intimacy (*F* (2,17) = 9.081, *p* < .01, *partial η* ^*2*^ = 0.348). Multiple comparisons revealed that this feeling was significantly stronger in the embodied touch condition compared to the no-touch condition (*p* < .01). Therefore, this result supports Hypothesis 1.

**FIGURE 3:**
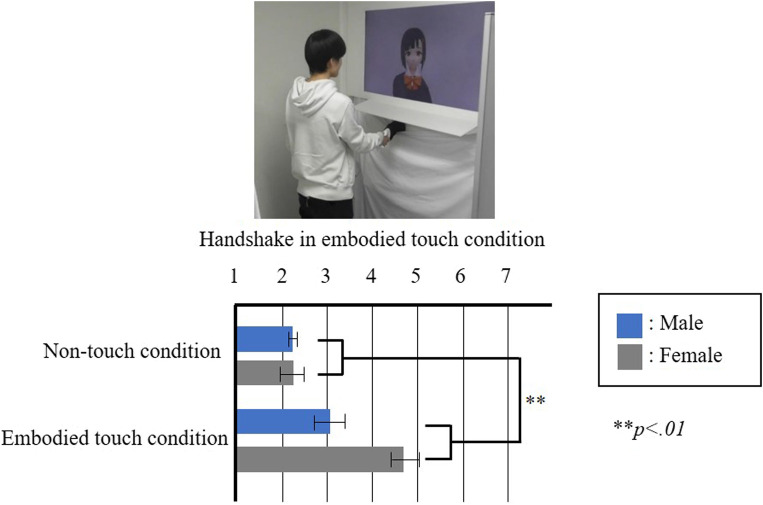
Results of the Experiment 1-b questionnaire “I felt that the drag-and-drop task was enjoyable.”

The results of Experiment 1-b are shown in Figure 3, wherein each box represents the mean value of the responses to each statement, and each bar represents the standard error of the mean value. In particular, we set two factors: one was contact factor and second was gender factor. The results were obtained with two-way factorial ANOVA followed by Bonferroni’s test, which noted a significant variation in the contact factor (*F*(1,24) = 4.260, *p* < .05, *partial η* ^*2*^ = 0.264). Furthermore, multiple comparisons implied the higher score of the embodied touch condition than the no-touch condition (*p* < .01). Overall, no significant variations were observed between the gender factors and interaction effects, thereby validating Hypothesis 1-b.

**FIGURE 4:**
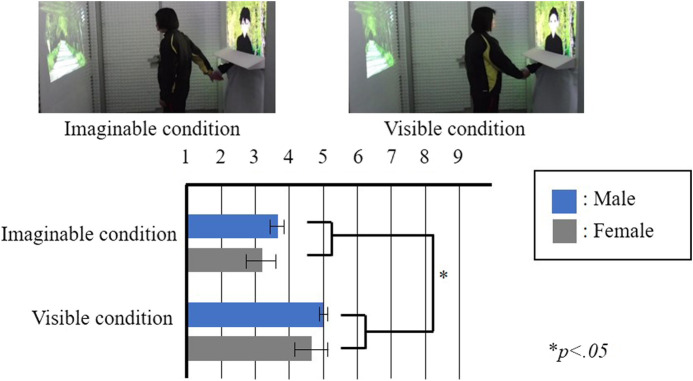
Results of the Experiment 2 questionnaire “I felt intimacy toward the partner.”

The results of Experiment 2 are shown in Figure 4, wherein each box represents the mean value of the responses to each statement and each bar represents the standard error of the mean value. The two factors were set, namely, visibility and gender. The results were calculated using two-way factorial ANOVA followed by Bonferroni’s test, which indicated significant variations in the visibility factor (*F* (1,10) = 6.914, *p* < .05, *partial η* ^*2*^ = 0.168). Thus, no significant variations were observed with factors of gender and interaction effects, which did not support Hypothesis 2.

**FIGURE 5:**
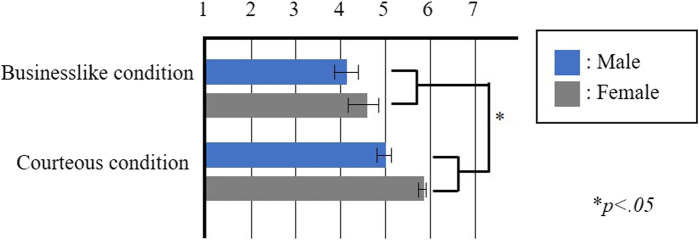
Results of the Experiment 3 questionnaire “I felt intimacy toward the partner.”

The results of Experiment 3 are shown in Figure 5, wherein each box represents the mean value of the responses to each statement and each bar represents the standard error of the mean value. We set two factors, namely, duration before releasing the participant’s hand and the gender factor. The results were calculated using two-way factorial ANOVA followed by Bonferroni’s test, which exhibited significant variations in the courteousness factor (*F* (1,18) = 5.0241, *p* < .05, *partial η* ^*2*^ = 0.218). However, no significant variations were observed in the gender factor and interaction effects. Therefore, the results support Hypothesis 3.

## 4 Deployment

Based on the experimental results, we organized a handshake event with “Hibiki Ao,” currently active as a virtual idol streamer, and their fans. Unlike the laboratory experiment, the participants in this event were real fans, and consequently, their intimacy toward the virtual idol would be noteworthy. We gathered feedback by observing fan behaviors.

### 4.1 Procedure

**FIGURE 6:**
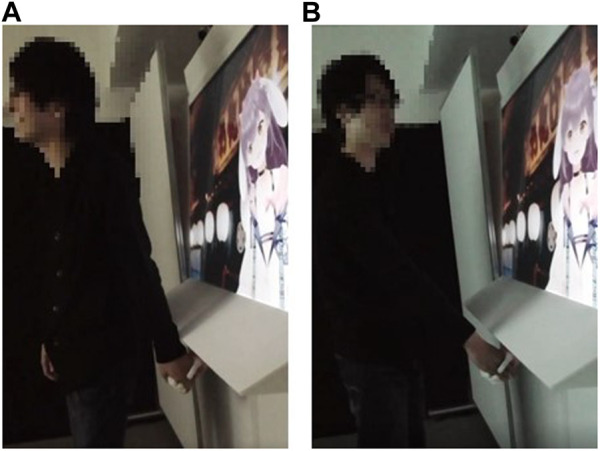
Images of participants holding hands with virtual idols **(A)** First half of event **(B)** Second half of event.

The participants held hands with the virtual idol during conversation for approximately 5 min. The event was segmented into two distinct halves. The first half included a handshake date while viewing the projector video on the screen, and the second half included a free talk with a handshake. Specifically, the handshake method varied between the first and second halves (Figure 6). In the first half, the virtual idol and participants were facing the same direction (similar to the imaginable condition in Experiment 2; Figure 6A), whereas in the second half, they were facing each other (similar to visible condition in Experiment 2; Figure 6B).

The flow of the event is stated as follows. First, we explained the flow of the event and informed the fans that we were recording throughout this event. After the explanation, we guided the participants toward the venue. Subsequently, we noted the participant’s name according to their preferred addressal by the virtual idols. Initially, the virtual idol and participants held hands looking at the same direction. After the guide exited the venue, the projector video and background music started, indicating the initiation of the handshake date. During the handshake date, the virtual idol and participants engaged in a conversation based on the video content. The duration of the video was approximately 2 min 30 s. After the video, the participants turned around to the virtual idol and held hands, but on this instance, they faced each other. Thereafter, a free talk started. After 2 min 30 s, a guide entered the venue and announced the end of the event. As the participants dispersed from the venue, we conducted an interview with informed consent to use the recorded video. The preliminary explanations and an interview were conducted outside the venue.

We analyzed the interview responses and the transcript of the interactions between the participant and virtual idol. At the interview, we asked participants to rank the pleasant instances of the event. Accordingly, we prepared four ranking items: “a conversation experience,” “a dating experience,” “a handshake experience,” and “others.” For the transcript, we transcribed the characteristics of the scene occurring at the event. The analysis with the transcript is a method of ethnomethodology that can qualitatively analyze the human communication. By noting the utterances and physical behaviors in chronological order, the analysis proceeded based on the order and timing.

Overall, 21 male fans of the virtual idol attended the organized event, among which certain individuals participated on multiple instances, and the experience was conducted 27 times in total.

### 4.2 Results

Based on the interview, the participants ranked their pleasant experience at the event. The answers from the rankings were only one time and we asked after the first time of experience. The results are presented in Tables 2 and 3. The distribution of the ranked experiences of the fans is presented in Table 2, wherein numerous fans placed the conversation experience at the first rank. In particular, no one ranked the handshake experience as third, and no one answered the item “others.” To analyze this ranking, we confirmed the number of participants answering the patterns of the ranking (Table 3). As such, three patterns could be observed from the responses of the participants.

**TABLE 2:** Ranking of event experience.

	First	Second	Third
Conversation experience	13	2	6
Handshake experience	8	13	0
Dating experience	0	6	15

**TABLE 3:** Number of participants answering the patterns of each ranking.

Patterns of the ranking	Number of responses
First: Conversation experienceSecond: Handshake experienceThird: Dating experience	13
First: Handshake experienceSecond: Dating experienceThird: Conversation experience	6
First: Handshake experienceSecond: Conversation experienceThird: Dating experience	2

### 4.3 Transcript

**FIGURE 7:**
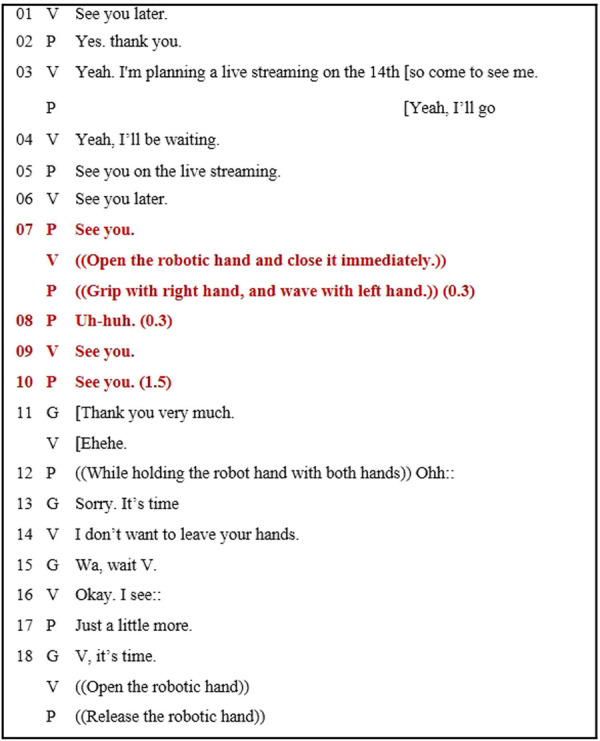
Transcript of virtual idol behaving courteously with the participant. V refers to the virtual idol, P denotes the participant, and G indicates the guide. The numbers in parentheses (*e.g* (0.0)) indicate the time of pause. Square bracket [indicates no “gap” between the two lines. (( )) describes the actions of the individuals.

To analyze the recorded video, we used a transcript of the conversation and transcribed the interactions between a participant and virtual idol to correspond the word order and meanings between the original language and English. The end-segment of a conversation between the virtual idol and a participant is depicted in Figure 7, wherein the red text denotes the important conversations. Based on the transcript, the virtual idol suggested ending the conversation (line 1), and in response, the participant accepted the suggestion (line 5). Generally, individuals release their hand at this instant. However, the participant and virtual idol retained their grasp and continued conversing for ∼2 s (from lines 7–10, red highlighted portion of transcript). Thereafter, the guide forcibly announced the end of event (line 11). Participants attempted to extend the event to the greatest possible extent (line 17). In this event, the virtual idol was conscious and did not release the participant’s hand until guided. Thus, the virtual idol courteously treated the fans, and the participant accepted the end of the event in conversation, but he did not accept it in his action. Possibly, this occurred because the virtual idol behaved courteously with the participant.

**Table:** 

01	V	See you later
02	P	Yes. thank you
03	V	Yeah. I’m planning a live streaming on the 14th [so come to see me
	P	[Yeah, I’ll go
04	V	Yeah, I’ll be waiting
05	P	See you on the live streaming
06	V	See you later
**07**	**P**	**See you**
	**V**	**((Open the robotic hand and close it immediately.))**
	**P**	**((Grip with right hand, and wave with left hand.)) (0.3)**
**08**	**P**	**Uh-huh. (0.3)**
**09**	**V**	**See you**
**10**	**P**	**See you. (1.5)**
11	G	[Thank you very much
	V	[Ehehe
12	P	((While holding the robot hand with both hands)) Ohh:
13	G	Sorry. It’s time
14	V	I do not want to leave your hands
15	G	Wa, wait V
16	V	Okay. I see:
17	P	Just a little more
18	G	V, it’s time
	V	((Open the robotic hand))
	P	((Release the robotic hand))

## 5 Discussion

According to Experiment 1, we tested the effectiveness of a robotic handshake by comparing the proposed method that reproduces the handshake of a Japanese idol with a conventional method of face-to-face interaction with the virtual idol. According to the free description in Experiment 1-a, there were comments such as feeling the tactile sensation of the hand during the handshake and perceiving the virtual idol as human-like. We consider that perceiving the virtual idol as human-like raised the sense of intimacy towards the virtual idol. According to the interviews, the participants realized that they were not touching the virtual idol’s hand, because the high-five was touched by the flat display. In addition, as participants touched the robotic hand in the embodied condition, the feeling of touching the virtual idol’s hand was strengthened. Thus, embodying a part of the virtual idol enhanced the sense of intimacy. As observed from the results of Experiment 1-b, the participants perceived the drag-and-drop task as an enjoyable experience under the embodied touch condition. Therefore, we can consider that the handshake enhanced the social presence of the virtual idol and its reality. These reasons enhanced the feeling of joy. Certain participants remarked that they were surprised by the temperature and softness of the robotic hand (one male and two females in embodied touch condition). The size of the robotic hand remained the same throughout the experiment. Presumably, the impression of the robot hand varied because the male’s hands are larger than female’s hands, implying that the females were more responsive to the robotic hand’s movements than the males. This reason possibly explains the higher score of the females under the embodied touch condition, although no significant variations were observed in the gender factor. In addition, fans participating in the event exhibited similar reactions as participants in the experiment. The fans commented that handshake with the virtual idol provided them happiness, surprise, and their heart were beating. Therefore, the fans exhibited a positive impression of shaking hands with the virtual idol. Based on the results stated in Table 2, several fans ranked the conversation experience as the best. In contrast, eight fans ranked the handshake experience as the best, and none ranked it at third. However, six fans ranked the conversation experience as third. Note that the fans participated in this event to engage in a conversation with the virtual idol rather than shaking hands with it. Regardless, all the fans were satisfied after shaking hands with the virtual idol. Thus, the handshake experience was more effective than the fan’s expectation.

Based on the results of Experiment 2, the participants experienced more intimacy toward the virtual idol in the visible condition than the imaginable condition. We expected that the boundary between the image of the virtual idol and the robotic hand eluded the sense of reality. Although the participants could experience the presence of the virtual idol in the imaginable condition, the results were contrary to our expectations. Thus, participants who engaged in handshakes were more effective than those in the visible boundary conditions. This result was confirmed from the interview of the event. In the items ranked in Table 3, 15 fans ranked the conversation experience (viewing the virtual idol) higher than the date experience (cannot view the virtual idol) and six fans ranked opposite. Certain participants preferred the imaginable situation. The impression with visible/imaginable conditions varied with the relationship between the user and virtual idol. As the fans participated in the event with prior knowledge of the virtual idol, they could imagine the appearance of the virtual idol without viewing it while holding hands.

The results of Experiment 3 revealed that the courteous behavior increased the sense of intimacy toward the virtual idol. In this experiment, the duration between the end of the conversation and the release of the robotic hand by the participant varied. We considered that the feeling of intimacy increased but not with the touching duration, and the virtual idol maintained the handshake, which can be verified from the transcript. At the event, the virtual idol was preconfigured to behave courteously. As observed from the transcript in Figure 7, the fan did not release their hand even after the conversation had ended. More importantly, the virtual idol did not release the fan’s hand, which was in line with the fan’s intention. Consequently, the fan felt a warm-hearted response that increased the sense of intimacy. We considered that this courteous behavior modified their action. Regardless of the users being fans of the virtual idol, its courteous behavior increased the sense of intimacy toward it.

## 6 Conclusion

This study focused on handshake with virtual idols and clarified the effective form of shake hands with them. We observed that the handshake by the robotic hand increased the feeling of intimacy toward the virtual idol, which became enjoyable to respond to a request from the virtual idol. In our interface, a certain contradiction was observed between the display and robotic hand, *i.e.*, a virtual idol is presented as an image and its robotic hand acts as a physical embodiment. Based on the experimental results, we observed that even if this contradiction was visible, the appearance of the virtual idols during the handshake increased the feeling of intimacy to the virtual idol. Furthermore, the typical handshake method for idols, wherein the user’s hand was grasped after the conversation, increased the feeling of intimacy toward the virtual idol. This courteous touch behavior was effective regardless of the user feeling a favorable impression of the virtual idol.

Virtual idols are nonphysical fictitious idols. The proposed method involved physically shaking hands with them. To combine a handshake gesture with virtual idols, the simulation of the courteous behavior is essential, as observed with real idols. Therefore, the effect of embodying the hand of a virtual idol is necessary, including the effect of their visual contradictions. This study revealed that agents with special roles, like idols, can enhance a sense of familiarity towards them by imitating the behaviors of real idols. As virtual agents with various roles are expected to emerge in the future, we anticipate that the findings of this study regarding social touch that imitates human interactions will be applicable.

## Data Availability

The original contributions presented in the study are included in the article/Supplementary Material, further inquiries can be directed to the corresponding author.
